# *Baylisascaris procyonis* in California

**DOI:** 10.3201/eid1009.040034

**Published:** 2004-09

**Authors:** Laurel Moore, Lawrence Ash, Frank Sorvillo, O.G.W. Berlin

**Affiliations:** * University of California, Los Angeles, Los Angeles, California, USA

**Keywords:** letter, Baylisascaris procyoni, prevalence, raccoons

**To the Editor:** We read with interest the article of Roussere et al. on the distribution of *Baylisascaris procyonis* eggs in northern California communities (*1*). The widespread dissemination and high density of raccoon latrines in residential areas clearly pose potential health risks, particularly to young children.

While California has reported more cases of baylisascariasis than any other state, few published studies have reported on the distribution and prevalence of this helminth in the region. In 2001, we conducted a study to determine the presence of *B. procyonis* in the Santa Barbara area by examining roadkill raccoons recovered by animal control staff and stored in a refrigerated facility. On examination, the digestive tract from the stomach to the rectum was removed and tested for *B. procyonis* worms and eggs. Of 26 raccoons examined, 24 (92%, 95% confidence interval 75%–99%) were positive for *B. procyonis* infection. *B. procyonis* worms were found in 85% of the animals examined and eggs were found in 73%. Pet food was frequently found (43%) in the stomach contents of examined raccoons, indicating that such food was made accessible to these animals, either intentionally or inadvertently by residents.

*B. procyonis* has been identified along the central coast of California, which expands the known range of this helminthic zoonotic agent. This finding, coupled with other published studies, indicates that *Baylisascaris* may be prevalent throughout the state (*1**,**2*). Although our study was based on a small sample of selected raccoons, the high infection rate is cause for concern and indicates the potential for human exposure. A presumptive case of *B. procyonis* infection in an 11-month-old child was reported in Santa Barbara in 2003 (*1*).

Determining the distribution and prevalence of *B. procyonis* is necessary to inform local healthcare providers, public health authorities, and the public of the potential risk. Using road-kill raccoons is a relatively easy method for quickly assessing the presence of *B. procyonis* in a community. Also, this approach avoids trapping and handling live animals and allows stomach contents to be examined to determine where raccoons are feeding. Data from such assessments must be interpreted with caution, since they may not represent all raccoons in an area.
